# Supporting adolescent girls to stay in school, reduce child marriage and reduce entry into sex work as HIV risk prevention in north Karnataka, India: protocol for a cluster randomised controlled trial

**DOI:** 10.1186/s12889-015-1623-7

**Published:** 2015-03-25

**Authors:** Tara S Beattie, Parinita Bhattacharjee, Shajy Isac, Calum Davey, Prakash Javalkar, Sapna Nair, Raghavendra Thalinja, Gautam Sudhakar, Martine Collumbien, James F Blanchard, Charlotte Watts, Stephen Moses, Lori Heise

**Affiliations:** Department of Global Health and Development, London School of Hygiene and Tropical Medicine, 15-17 Tavistock Place, London, WC1H 9SN UK; Karnataka Health Promotion Trust, Bangalore, India; University of Manitoba, Winnipeg, Canada

**Keywords:** Child marriage, Sex work, *Devadasi*, Sexual debut, Adolescent, India, Gender, Social norms, Maternal and child health, HIV

## Abstract

**Background:**

Low caste adolescent girls living in rural northern Karnataka are at increased risk of school drop-out, child marriage, and entry into sex-work, which enhances their vulnerability to HIV, early pregnancy and adverse maternal and child health outcomes. This protocol describes the evaluation of *Samata*, a comprehensive, multi-level intervention designed to address these structural drivers of HIV risk and vulnerability.

**Methods/Design:**

The *Samata* study is a cluster randomised controlled trial that will be conducted in eighty village clusters (40 intervention; 40 control) in Bijapur and Bagalkot districts in northern Karnataka. The intervention seeks to reach low caste girls and their families; adolescent boys; village communities; high school teachers and school governing committees; and local government officials. All low caste (scheduled caste/tribe) adolescent girls attending 7^th^ standard (final year of primary school) will be recruited into the study in two consecutive waves, one year apart. Girls (n = 2100), their families (n = 2100) and school teachers (n = 650) will be interviewed at baseline and at endline. The study is designed to assess the impact of the intervention on four primary outcomes: the proportion of low caste girls who (i) enter into secondary school; (ii) complete secondary school; (iii) marry before age 15; and (iv) engage in sex before age 15. Observers assessing the outcomes will be blinded to group assignment. The primary outcome will be an adjusted, cluster-level intention to treat analysis, comparing outcomes in intervention and control villages at follow-up. We will also conduct survival analyses for the following secondary outcomes: marriage, sexual debut, pregnancy and entry into sex work. Complementary monitoring and evaluation, qualitative and economic research will be used to explore and describe intervention implementation, the pathways through which change occurs, and the cost-effectiveness of the intervention.

**Discussion:**

This is an innovative trial of a comprehensive intervention to improve the quality of life and reduce HIV vulnerability among marginalised girls in northern Karnataka. The findings will be of interest to programme implementers, policy makers and evaluation researchers working in the development, education, and sexual and reproductive health fields.

**Trial registration:**

ClinicalTrials.Gov NCT01996241. 16^th^ November 2013.

## Background

### Vulnerability of low caste adolescent girls in northern Karnataka

An estimated 22 million girls are married globally, with more than one half of south Asian girls married before the age of 18 [1]. Child brides, defined by UNICEF as married before age 18 years, usually have older partners and more frequent, less protected sex than their unmarried, sexually active peers [1-3]. Married girls typically face intense pressure to become pregnant; hence early marriage is usually accompanied by early child-bearing and increased risk of maternal and child morbidity and mortality [4-8]. In some settings they are at greater risk of HIV Infection [9]. Problems associated with pregnancy and childbirth are a leading cause of death for girls ages 15–19 worldwide [7]. Married adolescent girls tend to have limited or no social networks, restricted social mobility, little autonomy and little or no access to media and health messages [1,10]. In addition, child marriage frequently ends a girl’s education [11]. Little or no schooling is associated with extreme poverty and hunger [12]; gender inequality [13,14]; increased child mortality [15-17]; poor maternal health [18]; increased teenage pregnancy rates [19,20]; early sexual debut; child marriage [11,21]; increased fertility levels [18] and increased HIV infection [19,22]. Secondary school drop-out is also associated with higher levels of intimate partner violence [23,24].

Bijapur and Bagalkot are districts in northern Karnataka, characterised by high rates of poverty, unemployment, illiteracy and migration [25]. The primary source of employment is seasonal agricultural labour. A disproportionate share of adolescent girls are “missing” from their natal households, due to early transition into adult roles via marriage or entry into sex work [26]. Although prohibited by law, child marriage remains common, particularly among those from ‘low castes’ (defined here as scheduled caste and scheduled tribe), with 47.5% and 36.4% of low caste girls in Bagalkot and Bijapur districts, respectively, marrying before age 18 [27] (unpublished survey data, DHLS III surveys, Karnataka (2007–8).

Northern Karnataka is also home to the *Devadasi* tradition of sex work, a cultural practice that dedicated young girls to the temple Goddess “*Yellamma*” [28]. Once they reached menarche, dedicated girls became servants at local temples, often combining dancing and other artistic functions with provision of sexual services to the priests and (higher caste) temple patrons [29]. Nowadays, dedicated girls often practice commercial sex work without religious overtones [30]. Mapping and migration studies estimate that at least seventy-five brothels in the neighbouring state of Maharashtra, are run by madams from Bijapur and Bagalkot districts, who recruit new girls from impoverished families during festivals and other celebrations. Entry into sex work terminates girls’ education and sharply increases their HIV risk; FSWs under 20 years old are between two and four times more likely to be HIV-infected than FSWs who are older [31].

Pre-trial qualitative research suggests that a range of social-structural factors, including poverty and poor livelihood options, gender differences in parents’ expectations and aspirations for their children, and caste-related discrimination, contribute to girls having shorter education than boys, and perpetuate the traditions of underage marriage and sex work through the *Devadasi* tradition.

### Adolescent and gender focused interventions – the evidence to date

A global review of research and programmes that aim to increase school retention or decrease early marriage and/or entry into sex work found only a few programmes that have been evaluated, mostly in sub-Saharan Africa. These studies suggest that keeping girls in school can increase their age at first sex [32], increase their age at marriage [21,33], and reduce their risk of HIV infection [22]. In addition, conditional and unconditional cash transfers can increase school retention [34] and reduce HIV and STI rates [19], suggesting that interventions that focus on structural sources of vulnerability (such as poverty) can have multiple (health and education) benefits [35]. Nonetheless, there is little evidence as to what works best to reduce adolescent girls’ vulnerability in this setting.

## Methods/Design

### The samata intervention

The overall goal of *Samata* is to improve the quality of life of adolescent girls from vulnerable and marginalized communities in Bijapur and Bagalkot districts of north Karnataka, by supporting entry into and retention of adolescent girls in secondary education, and by delaying age at marriage and entry into sex work. Specifically, the intervention aims to bring levels of these outcomes among low caste girls in-line with those of all girls in Karnataka. The project comprises a comprehensive, multi-level intervention that works with key stakeholders (low caste adolescent girls and their families; adolescent boys; village communities; schools; school development and management committees (SDMC); and policy makers and policy implementers), to change social norms regarding gender, child marriage and girls’ education, as well as to link low caste families to government schemes that provide scholarships, bicycles and other incentives to support girls’ retention in school. A summary of the main intervention activities are detailed in Table 1. The intervention will be implemented in 119 intervention villages in Bijapur and Bagalkot districts in northern Karnataka, and will run for a period of three years (2015–2018).

**Table 1:** **Summary of intervention activities for project Samata**

**Stakeholder**	**Intervention activities**
Low caste adolescent girls	(i) Identify all low caste (scheduled tribe/scheduled caste) girls every year and track their situation.
	(ii) Develop individual plans for outreach and follow up
	(iii) Establish safe spaces in which girls meet, receive life skills training, and gain leadership skills
	(iv) Encourage attendance at special tutoring sessions designed to meet the needs of girls, particularly those who have fallen behind their peers.
Low caste families of adolescent girls	(i) Outreach workers conduct home visits and family meetings to sensitise parents around girl’s education, early marriage, and gender socialisation
	(ii) Map vulnerability in each low caste family
	(iii) Link low caste families to government schemes that provide material and financial incentives for educating girls.
Adolescent boys	(i) Recruit and train local mentors to deliver “Parivartan,”--a sports-based, life-skills and empowerment programme. This programme encourages critical reflection on gender norms, including attitudes around violence against women and ‘eve’ teasing (sexual harassment/abuse) of girls.
	(ii) Form Parivartan boys groups in each village and implement activities.
Village communities and leaders	(i) Use community meetings and street theatre to sensitise local communities to the importance of girls’ education and the consequences of early marriage
	(ii) Develop local champions to encourage communities to take action to retain girls in school.
School staff and governing committees	(i) Train and equip school staff and school development and management committee (SDMC) members to conduct gender analyses of the school environment and to design and implement plans to make schools more “girl friendly”.
	(ii) Train staff and SDMC members to track school attendance of adolescent girls
	(iii) Support and train school staff and committee members to develop leadership and career counselling programmes for girls
	(iv) Support and train school staff to develop policies that ensure the safety and participation of girls in school.
Policy makers and Policy implementers	(i) Advocate with local government to support the project by briefing them regularly
	(ii) Share project findings and learnings to advocate for replication of key strategies.

### Study aims

The study aims to assess the impact of project *Samata* on levels of (i) high school entry; (ii) high school retention; (iii) age at marriage; and (iv) age at sexual debut of low caste adolescent girls. In addition to these co-primary outcomes, we will conduct a survival analysis for marriage, sexual debut, pregnancy and entry into sex work and explore how the intervention has affected the school and the communities’ response to premature school drop-out, as well as the processes and causal pathways through which changes occur for the following secondary outcomes: social norms and attitudes related to girl’s education, gender roles, early marriage, and sexual harassment; girl’s sense of self-esteem and self-confidence; expansion of girl’s networks; girl’s experience of harassment in the past six months; and girl’s entry into sex work.

### Study design

The study will employ a cluster-randomized controlled trial design. A cluster-randomized design was chosen because the intervention is at a cluster level, i.e. many components of the intervention will not be delivered to specific individuals, but to the schools and the communities where the individuals study and reside. All high-schools in Bijapur and Bagalkot districts have been enumerated (n = 1075). For practical and logistical reasons, schools with the following criteria were excluded from the study: schools in urban areas (n = 319); schools that are private and unaided by the government (n = 87); schools that are only for boys (n = 13); and schools in villages where the total number of low caste (scheduled caste/scheduled tribe) girls enrolled in 8th standard was less than 10 (n = 431). This left 225 high-schools eligible for the study, located in 121 village clusters; a ‘village cluster’ comprises one ‘main’ village with one or more eligible high-schools plus the surrounding ‘feeder’ villages, which do not contain a high-school but have low caste children living there who attend the high-school in the ‘main’ village. The village cluster was chosen as the unit of randomization instead of the school so as to minimize contamination between schools in the same village.

### Power calculations and selection of villages

Sample size calculations were based on estimates of: (i) the harmonic mean cluster size; (ii) the refusal/loss to follow-up rate; (iii) the between cluster variation (κ); and (iv) the estimated levels in the control villages of the following four co-primary outcomes:

1. Proportion of those enumerated in 7^th^ standard (the study cohorts) who enter into standard 8.
2. Proportion of those in the study cohorts who complete standard 10.
3. Proportion of girls in the study cohorts who are married before age 15.
4. Proportion of girls in the study cohorts who experience first sexual intercourse before age 15.

The expected effect sizes were guided by the aim of the intervention, i.e. to bring the primary outcomes in-line with all girls in Karnataka, and are detailed in Table 2. We used the prevalence of these outcomes from the most recent district and state wide surveys to estimate plausible effect sizes. We have sized the trial to detect a risk ratio of 0.70 for transition from standard 7 to 8 among low caste girls in the intervention villages compared with control villages. This is based on a state-level dropout rate among low caste girls of 9% during higher primary grades, compared to 5.6% among all girls, corresponding to a risk ratio of RR = 0.62 [36]. We have sized the trial to detect the weaker RR of 0.70, as district level data were not available, and it is plausible that the intervention will not be able to fully bring low caste drop-out during transition to the average among all girls in Karnataka. It is assumed that retention in school until the end of 10^th^ standard will be harder to influence than transition from standard 7–8, as the barriers to staying in school increase over time. For this outcome, we have assumed a minimum detectable effect size of RR = 0.75-0.70, based on a state-wide school drop-out rate from standard 8 to standard 10 of 22.5% among low caste girls, compared to 16.5% among all girls, corresponding to a RR of 0.73 [36]. For age at marriage and age at sexual debut, we have assumed a minimum detectable effect size of RR = 0.75, based on a district-level ‘marriage before age 15′ rate of 25.9% among low caste girls compared to 19.3% among other girls (Unpublished data, District Level Household and Facility Survey (DLHS-3), 2007–8, Government of India). We aim to decrease the proportion married before age 15 by 25-30% (bringing the proportion in-line with all girls), corresponding to a risk ratio of 0.75-0.70. We have assumed the proportion who have sexual intercourse before age 15 is equal to the proportion who marry before age 15, and therefore also aim to decrease the proportion who have sexual intercourse before age 15 by 25-30%.

**Table 2:** **Parameters of the sample size calculation: expected effect sizes**

**Parameter**	**Derivation**
Harmonic mean cluster size	Based on an enumeration carried out in the 225 high schools located in 121 villages by KHPT. Approximately 15 SC/ST girls are available at each school. Therefore, over the two years, we have assumed a mean of 30 girls per cluster.
Refusal/loss to follow-up	Refusal and loss to follow-up are expected to be low, approximately 5-10% (14 girls per cluster per year - > 28 at endline)
Between cluster variation (k)	The between cluster variation in the outcomes is not known. Therefore, we have reported sample sizes for 0.15, 0.2, and 0.25.
**Control arm proportions**	
Proportion transitioning 7-8^th^ (actualized in terms of the proportion dropping out between 7^th^ and 8^th^)	We calculate cluster numbers for a range of drop-out proportions, from 9% (the State level drop-out proportion among SC/ST girls) to 17% (the dropout rate among all girls in Bijapur district [36]. These are conservative estimates.
Proportion completing 10^th^ (actualized in terms of the proportion dropping out between 7^th^ and 10^th^)	30-40% of girls drop out before standard 10 [36].
Proportion married before age 15	The district Level Household Surveys (DLHS-2007-08) collected data on age at marriage and has a reasonable sample size in each district. About 21% of women aged 18–25 married before the age of 15 years. A higher percent of SC/ST women were married compared to others (25.9% vs. 19.3%)^1^. Therefore the proportion is likely to be around 25%
Proportion sexual intercourse before age 15	Data from IBBA surveys among FSWs conducted in Belgaum district found 44.7% of FSWs reported their first sex was before the age of 15 years^2^. Given 25.9% of SC/ST women married before the age of 15 years, the proportion who had their first sex before 15 years will be a number above this, but within 44.7%. The Polling Booth Surveys conducted in the rural areas of these districts in 2011 found that 8% of unmarried females (15–24 years) had ever had sex, whereas 5.3% of married females (15–24 years) had sex before marriage in Bijapur district^3^. Therefore the proportion who had sex before 15 years is likely to be a number above 30%.
**Effect (risk ratio)**	
Proportion transitioning 7-8^th^	We have reported a range of Risk Ratio minimum detectable effect sizes of 30-40%. This is considered to be the most likely of the outcomes to be influenced by the intervention
Proportion completing 10^th^	We have reported a range of Risk Ratio minimum detectable effect sizes of 20-25%. The lower effect size compared to outcome 1 reflects the likelihood that this will be harder to influence, as the barriers to staying in school increase over time
Proportion married before age 15	The intervention will target marriage directly through school and home based interventions. We aim to decrease the proportion married before 15 by 25-30%.
Proportion sexual intercourse before age 15	The intervention will target sexual debut directly through school and home based interventions. We aim to decrease the proportion married before 15 by 25-30%.
Type 1 error	We have set this at the 5% level
Power	We have set this at the 80% level

^1^Unpublished, District Level Household and Facility Survey (DLHS-3) data, 2007–8, Government of India. ^2^Unpublished female sex worker, integrated behavioural biological assessment (IBBA) survey data, Belgaum District, 2010, Karnataka Health Promotion Trust. ^3^Unpublished general population, polling booth survey data (PBS), 2011, Karnataka Health Promotion Trust.

We are uncertain about many of the parameters underlying the sample size calculation, especially the value of k – the between village variation in the outcomes. Therefore, we explored the relationship between the number of clusters and the value of K for a range of realistic estimates of the above parameters, shown in Table 3. In a large proportion of the scenarios shown, we will have sufficient numbers of clusters to detect an effect. However, there are plausible conditions where as many as 54 clusters per arm would be required to detect an effect.

**Table 3:** **Number of clusters required in each arm under different assumed conditions**

**Transition from 7-8th standard**				**Retention in school**				**Proportion married before age 15**				**Proportion having had sexual intercourse before age 15**			
**Control p**	**% reduction**	**K**	**# clusters per arm**	**Control p**	**% reduction**	**K**	**# clusters per arm**	**Control p**	**% reduction**	**K**	**# clusters per arm**	**Control p**	**% reduction**	**K**	**# clusters per arm**
17%	30%	0.15	30.4	40%	20%	0.15	28.3	35%	25%	0.15	20.8	35%	25%	0.15	20.8
		0.2	32.7			0.2	33.9			0.2	24.3			0.2	24.3
		0.25	35.6			0.25	**41.2**			0.25	28.7			0.25	28.7
	33%	0.15	24.9		23%	0.15	21.3		30%	0.15	14.4		30%	0.15	14.4
		0.2	26.7			0.2	25.5			0.2	16.7			0.2	16.7
		0.25	29.1			0.25	30.8			0.25	19.6			0.25	19.6
13%	30%	0.15	40	30%	20%	0.15	38.8	30%	25%	0.15	24.6	30%	25%	0.15	34.1
		0.2	**42.3**			0.2	**44.4**			0.2	28			0.2	37.5
		0.25	**45.2**			0.25	**51.7**			0.25	32.4			0.25	**41.9**
	33%	0.15	32.7		23%	0.15	29.1		30%	0.15	16.9		30%	0.15	23.4
		0.2	34.5			0.2	33.3			0.2	19.2			0.2	25.6
		0.25	36.9			0.25	38.6			0.25	22.1			0.25	28.5
9%	35%	0.15	**41.7**	20%	23%	0.15	**44.8**	20%	25%	0.15	37.7	20%	25%	0.15	37.7
		0.2	**43.3**			0.2	**48.9**			0.2	**41.1**			0.2	**41.1**
		0.25	**45.4**			0.25	**54.2**			0.25	**45.5**			0.25	**45.5**
	40%	0.15	31.3		25%	0.15	37.7		30%	0.15	25.8		30%	0.15	25.8
		0.2	32.4			0.2	**41.1**			0.2	28			0.2	28
		0.25	33.9			0.25	**45.5**			0.25	31			0.25	31

This table shows the number of clusters required in each arm to detect a range of potential effect sizes. Conditions where more than 40 clusters would be required per arm are highlighted in bold.

For our main power calculations we have assumed a modest refusal rate and loss to follow-up rate of around 7%. We have included conservative estimates of the control outcome proportions, and reported calculations for a range of K from 0.1 to 0.25 (Table 3). The selection of 80 village clusters with a harmonic average of 30 low caste girls in 7^th^ standard per cluster, enrolled in two sequential cohorts of 15 girls per year, should be sufficient to detect the effect estimates for the four co-primary outcomes. With minimal loss to follow-up, and a minimum of 28 girls per cluster followed-up at endline, we will have 80% power to detect a 33% reduction in the risk of not making the transition from 7-8^th^ standard (i.e. a risk ratio of 0.67 between the arms), and a 25% reduction in the risk of not remaining in school until the end of 10^th^ standard (i.e. a risk ratio of 0.75 between the arms).

### Selection of villages

**Figure 1:**
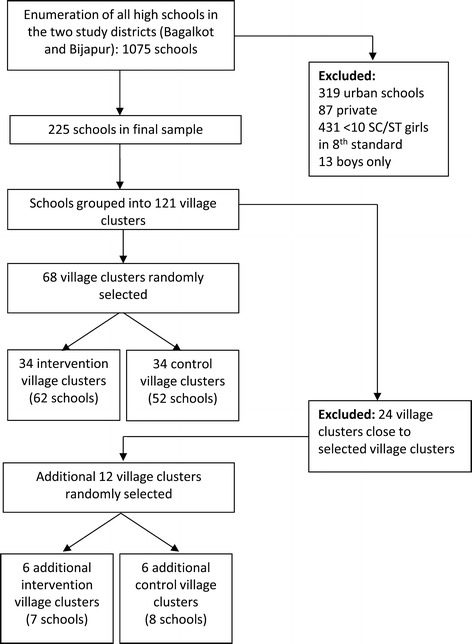
**Consort diagram for** ***Samata*** **trial.**

80 village clusters were selected using a systematic random sampling method from the sample frame of 121 village clusters, using STATA 11. The selected village clusters were allocated randomly to either receive the intervention immediately (experimental condition) (n = 34), or at a later date (waitlist control group) (n = 34) (using STATA 11). We notified the selected village clusters of their selection into the study and have begun outreach and advocacy work with community leaders in the intervention villages. After allocation, we realised that there were 12 village clusters where there was a possibility of contamination, because these villages were contiguous with a village cluster in a different arm of the study. To compensate for potential loss of statistical power due to contamination, we selected an additional 12 village clusters (6 intervention and 6 control arm) for inclusion in the study as follows: of the 53 village clusters remaining in the original sampling frame of 121 village clusters, we excluded 24 village clusters due to their close proximity to villages that had already been selected for the study. From the remaining 29 village clusters in the sampling frame, 12 village clusters were randomly selected to be included in the study and these were randomly allocated to intervention and control arms using STATA 11. Thus the trial will be implemented in 80 village clusters (40 in each study arm), encompassing 296 villages (119 intervention and 177 control arm) and 129 high schools (69 intervention schools and 60 control schools) (Figure 1).

### Study participants

The study population will be all low caste girls (defined as belonging to scheduled caste or scheduled tribes) who have completed 7th standard. Most of them will be 13 years of age. We estimate that approximately 2,400 low caste girls (aged 13 years) will be enrolled in the trial in two cohort waves one year apart: 1,200 low caste girls in year one (600 control and 600 intervention arms) and 1,200 low caste girls in year two (600 control and 600 intervention)) (Table 4). Each low caste adolescent girl enrolled in the trial will be profiled and the main decision maker in her family will be identified and invited to participate in the study at the family level. All high-schools in the 80 study village clusters will be profiled and line-lists provided of all staff and school development and monitoring committee (SDMC) members. Each principal, 2 SDMC members and 2 teachers (1 male and 1 female) per school will be randomly selected for participation in the study. Adolescent boys (from the low caste community neighbourhood) (n = 20) will also be recruited purposively for qualitative interviews.

**Table 4:** **Summary of sequential cohort study design**

	**June 2013**	**June 2014**	**May 2016**	**May 2017**
Intervention site, cohort 1	600 girls who have completed 7^th^ standard and their families		Follow-up interviews	
Control site, cohort 1	600 girls who have completed 7^th^ standard and their families		Follow-up interviews	
Intervention site, cohort 2		600 girls who have completed 7^th^ standard and their families		Follow-up interviews
Control site, cohort 2		600 girls who have completed 7^th^ standard and their families		Follow-up interviews

### Interventions and comparisons

Girls in the control arm (40 village clusters) will be provided with the standard level of support as currently provided by the government and other NGOs. Project staff will visit each cohort member every 6 months so as to minimise loss to follow up at the endline survey. Where girls have migrated as a result of family migration or marriage, information regarding the location of migration will be recorded and kept so that the interviewers can go to their new location to administer the questionnaire at the endline survey.

Girls in the intervention arm (40 village clusters), along with their families, adolescent boys, village communities and their leaders, and staff and SDMC committee members at government-run high-schools, will receive the intervention described in the section above.

### Research methods

The trial has four main components:

1. A quantitative assessment involving two sequential cohort studies, one initiated in year 1 and another initiated in year 2, with low caste girls (n = 2400), their families (n = 2400), high-school staff and SDMC members (n = 650). The questionnaires will be administered at baseline and endline.
2. A qualitative assessment documenting the process of implementation. Longitudinal case studies, using participatory life-line methods, will be conducted annually with low caste girls (n = 40) to investigate how *Samata* has affected the girls and their families in addressing school dropout, early marriage, sexual debut and entry into sex work. In-depth interviews will be conducted at baseline and endline with adolescent boys (n = 20) to examine how the intervention has affected attitudes and norms around gender, education, marriage and violence.
3. A monitoring and evaluation system to monitor the intervention activities at the school and community levels. An individual tracking system that tracks individual girls will be developed to monitor inputs, outputs and certain outcome-level indicators by schools. The project will facilitate schools to track girls from marginalized communities during the project period to monitor school achievement and ensure efficient identification of drop-outs, so that the issues related to dropping out can be effectively and quickly addressed. At the community level, project activities (such as linkages to government schemes, school-to-community contact programs, support for tutorials/remedial classes with adolescent girls and their families), will be monitored.
4. Economic analysis to measure the costs, benefits and cost-effectiveness of the project.

### Analysis

#### Assessing baseline balance

We will present the level of balance at baseline by summarising important measured variables by arm, and will assess the magnitude of any differences. We will represent the precision of the summaries with 95% confidence intervals that account for clustering. We anticipate that the trial will be balanced as we have a relatively large number of clusters and the schools are all government schools of similar character.

### Primary analysis

We plan to conduct our primary analysis with simple between-arm comparisons in the co-primary outcomes of entering 8th standard, completing tenth standard, being married before age 15 and having sexual debut before age 15. In the course of the analysis, we will present simple cluster summaries for the primary outcomes, and report the empirical value of *k* with updated power calculations. Given the large number of clusters, and to increase statistical efficiency, individual-level logistic regression models will be used to estimate differences between intervention and control arms [37]. We will use random effects to account for the clustering by village, and include dummy variables for the strata. We will also estimate effects adjusting for cluster-level or individual-level variables that appear to be imbalanced at baseline. Stata 11 will be used for all analyses.

### Secondary analysis

We will conduct a survival analysis for marriage, sexual debut, pregnancy and entry into sex work. We will conduct sub-group analyses as follows: (i) the two cohorts of girls; (ii) per district; (iii) for non-devadasis and (iv) for socio-economic strata. For each sub-group analysis, we will test for interaction with the complete model, and report the interaction test p-values. We will explore differences between the arms of a range of parameters measured, using the questionnaires of the girls and their families.

### Qualitative data analysis

The qualitative data will be transcribed and translated into English. Qualitative analysis packages (eg., NVivo9, ATLAS TI) will be used to identify key issues and themes emerging over time.

### Process evaluation analysis

In the intervention arm, schools will use the monitoring system to track all participants on specific input, output and outcome indicators, as part of the intervention. These will include recording all services provided to each participant and their family, school attendance, school drop-out, marriage, academic scores, and mobility (for example due to migration, marriage or sex work). In the control arms, so as not to influence the outcomes through the effect of direct observation and data collection, the monitoring system will be limited to tracking only a few outcome indicators (school drop-out and marriage).

The monitoring data will be analysed and used to understand school attendance, drop-out rates, the proportion of girls marrying and the quality of education (academic scores). A unique identifier will be used for each participant to enable triangulation of the outcomes of interest between monitoring and survey data.

### Economic evaluation

An economic evaluation of the intervention will be conducted, from a provider perspective. This will follow established evaluation approaches, including both the start-up and operational costs of the intervention delivery, and valuing both the monetary and non-monetary inputs into the intervention delivery.

### Ethical considerations

We consider that the study merits a randomized design, as the intervention is novel and untested, and we do not know if it will be successful or not. The intervention attempts to address the social-structural drivers of school drop-out, early marriage and entry into sex work, and thereby reduce vulnerability to HIV, in the presence of multiple contextual factors and we feel there is sufficient equipoise about the impact to warrant an RCT design. If the intervention is successful, we plan to implement *Samata* in the 40 control communities at the end of the trial, pending funding. The design has been finalized after many rounds of discussions with stakeholders at different levels. The design has attempted to take into consideration the existing structures, the regulatory environment and the cultural practices, and perspectives of major stakeholders, including the Karnataka government.

Interviews will be conducted in *Kannada* (the local language) in private settings in a sensitive and non-judgmental manner. Since some of the participants will be minors, we will seek both informed consent from the girl’s parent or legal guardian (if she is unmarried) and informed assent of the girls. Many of the parents in this region are illiterate and reluctant to sign documents. Therefore we will accept either written consent or verbal, witnessed consent. We will seek independent informed consent from girls who are married and living with their husband. Girls who are illiterate will be asked to assent in front of a literate witness of their choice (but not a parent or guardian). The girl will acknowledge her assent with a thumbprint and the witness will sign the document. The *Kannada*/English version of the consent/assent form will be given to the participants to read and will also be read out and explained before the beginning of the interviews. As part of the consenting/assenting procedure, participants will be assured that their participation is voluntary, and their decision to participate will not affect any benefits they receive from the school or the intervention.

The main potential area of distress for girls relates to answering sensitive questions around sexual activity, pregnancy, harassment or sexual coercion. To limit embarrassment and encourage disclosure, sensitive questions will be asked through an anonymous pen and paper questionnaire, administered at the end of the face to face interview. If girls cannot read, the interviewer will read the questions aloud and allow the girls to tick the appropriate box privately on the paper. After completing the sensitive questions, girls will fold the paper ballot and place it in a see-through bag with the answers from many other respondents. The questionnaire asks about eve teasing, transactional sex, sex work and sexual coercion, but it does not collect information on who is responsible for harassing or sexually abusing the girl. Interviewers will be trained how to respond if the girl becomes upset while answering these questions or at any other time during the interview, including offering referral and transport to local support and counselling services.

Anonymity will be maintained by using proxy names on qualitative interview transcripts and in any publication that quotes a participant. Quantitative survey data will be anonymised and a unique participant identification number will be used to link survey rounds. The identity of the participants and the information shared by them will not be revealed to anyone who does not work in the research study. Unique identifying numbers will be used to identify the questionnaires; no identifying names will be entered with the computer data. Paper copies of the data will be kept for 5 years after completion of the end of the study before being shredded.

In order to ensure equity to all schools enrolled in the trial, we are applying for funding to enable the school-based interventions to be provided in these schools at the end of the intervention period if the trial indicates that the intervention was effective.

This study has been approved by the Institutional Ethical Review Board of St. John’s Medical College, Bangalore, India, and the Observational/Interventions Research Ethics Committee of the London School of Hygiene and Tropical Medicine and the University of Manitoba.

## Discussion

The *Samata* study is, to our knowledge, the first cluster randomised controlled trial to assess the impact of a gender-focused, comprehensive structural intervention on secondary school drop-out, child marriage and entry into sex work in Asia, and one of the first globally. It uses a rigorous methodology designed to minimise (unmeasured) confounding and several forms of selection and measurement bias.

We aim to keep selection bias to a minimum through the randomization of the study villages and the selection of *all* low caste (scheduled caste and scheduled tribe) girls enrolled in 7th standard for participation in the study. However, although district and state level surveys suggest that the majority of school-drop outs occur between 7th and 8th standard, the most vulnerable girls may have dropped-out before 7th standard and not be included in the study, which in turn could lead to an underestimation of study effect. In addition, recruitment bias and selective loss to follow-up due to migration for work, marriage or sex work of those at highest risk of the study outcomes, could lead to a bias in the cohort resulting in underestimation of the study impact. We plan to address this issue at baseline by repeatedly visiting villages to interview study participants who are ‘missing’, until they have returned from migration. In addition, we plan to minimise loss to follow-up by visiting all participants at least 6 monthly, and collecting information on the whereabouts of those who have migrated to help with tracking them for interview at endline.

Reporting bias, particularly around sensitive topics such as sexual relationships, marriage, pregnancy, sex work, and experiences of sexual harassment and violence, may lead to an underestimation of these outcomes, reducing the power to detect a difference between the study arms. We will attempt to minimise this potential bias by conducting rigorous training of experienced research assistants to administer the questionnaire, by reassuring participants around confidentiality during the assent process, and by including a self-completed section at the end of the questionnaire which contains these sensitive questions. This method has proven successful in reducing reporting bias in other studies [38]. In addition, we have included some questions (such as number and occupation of siblings, recent migration and school absenteeism) in both the family and girl questionnaires, to enable triangulation of data. We have sized the study using data collected from face-to-face questionnaires, which may have underestimated the true prevalence of sensitive behavioural parameters because of social-desirability bias. Recall biases are expected in some questions in the survey related to age of menarche, frequency of home visits, number of days missed from school, etc. We have attempted to minimise these by the careful design of questionnaires (for example by using relatively recent time-frames for recall, where possible), and rigorously training the interviewers to build a good rapport with the respondents and to encourage respondents to correctly recall responses. Interviewer bias will be addressed to a large extent by rigorous training of the research investigators and by blinding of the interviewers to the study arms.

The ‘Hawthorne’ effect, whereby participants modify their behaviour simply because they are being observed [39], could lead to an underestimation of the impact of the intervention. We have designed the monitoring and evaluation framework to try to minimise contact with the control communities, to try to reduce this effect. However, field staff have reported that some teachers in the control arm noted down the various schemes available for low caste girls during the baseline interviews, presumably to link girls in their communities with these schemes.

The lack of biological outcomes (such as HIV, STIs and pregnancy related outcomes) is a limitation of this study, but the number of participants needed to detect a difference in these relatively rare outcomes would have been prohibitive. The intervention is time and labour intensive, as it involves all major stakeholders and covers a vast geographical area. To ensure quality as well as scale, we have employed a robust monitoring and evaluation system, data from which will be scrutinized monthly to provide feedback and influence the intervention process. In addition, the intervention team has extensive experience in implementing large-scale, effective intervention programmes in this area, under the *Avahan* intervention in India [40]. If the intervention proves effective in achieving the primary outcomes, the impact, process and costing evaluations will be important in assessing which components were particularly important and cost-effective and should be included in intervention replication elsewhere.

In conclusion, despite widespread efforts to improve adolescent girl retention in secondary school and reduce child marriage and entry into sex work at a young age, this is one of the first randomised controlled trials conducted on these issues. The trial will provide important findings which can be used to inform policy, both in the education, as well as in the development and sexual and reproductive health fields. The trial will also provide evidence on the feasibility of the approach of addressing distal and proximate drivers of vulnerability among adolescent girls in this setting.
